# Chartering new waters of biofilm ecology and evolution

**DOI:** 10.1038/npjbiofilms.2015.2

**Published:** 2015-03-25

**Authors:** Tom J Battin

**Affiliations:** 1 Ecole Polytechnique Fédérale Lausanne (EPFL), Lausanne, Switzerland

Biofilms dominate microbial life in most natural, engineered and medical systems, and they have orchestrated key biogeochemical cycles on Earth over billions of years.

As the formation of biofilms goes through distinct stages, biofilm research itself has evolved through various stages since the early work by Claude ZoBell. Advances made *en route* were critical and greatly benefited human well-being and public health—or they were simply to the pure delight of science. No doubt, Bill Costerton, having realised that most microorganisms in harsh alpine streams dwell in biofilms, has heralded a new era of biofilm research and his brilliant work has illuminated the darkness of the biofilm mode of life.

Driven by the humble wish to serve humanity, entire cohorts of microbiologists devoted their careers to biofilms involved in infections and other diseases.

Their gunship soon became the ‘flow cell’. In an attempt to understand and predict the complexity of single bacterial populations and mixtures thereof, they produced an impressive body of knowledge still often driven by the classical Koch postulate. The genetic toolbox enabled them to decipher key regulative pathways and to gain mechanistic knowledge of basic bacterial behaviour. The advent of imaging techniques, microelectrodes and modelling uncovered the full beauty but also the full complexity of biofilms—even of the simplest among them.

What have we learned from the lesson? That we need to comprehend biofilms as ecological communities that steadily interact with the environment that they inhabit, and that we need to better root biofilm research in ecological and evolutionary sciences.

Biofilms are jungles of microbial life, even with a biodiversity equal or superior to the biodiversity of plants in a tropical rainforest or of fish in the ocean. To understand the massive complexity of these communities, we must opt for a holistic approach that integrates biology, physics and chemistry—and let us not forget process coupling and feedbacks.

Merging novel technologies from life sciences and engineering offers unprecedented opportunities to address future questions in biofilm ecology and evolution. These questions are fundamental to understand the mechanisms that make biofilms so successful over evolutionary times and potent factors in Earth processes. Only then we will be able to exploit the biomechanics, biodiversity and functioning of biofilms, and to bioengineer them to systems that provide goods and services in a rapidly changing world.

May *npj Biofilms and Microbiomes* serve as the prime outlet to share the most exiting research on biofilms, and to pave the way towards ground-breaking strides in biofilm ecology and evolution.

